# Deep Learning-Assisted Cactus-Inspired Osmosis-Enrichment Patch for Biosafety-Isolative Wearable Sweat Metabolism Assessment

**DOI:** 10.3390/bios15120790

**Published:** 2025-12-01

**Authors:** Yuwen Yan, Ting Xiao, Miaorong Lin, Wenyan Yue, Jihan Qu, Yonghuan Chen, Zhihao Zhang, Jianxin Meng, Dong Pan, Fengyu Li, Bingtian Su

**Affiliations:** 1 Su Bingtian Center for Speed Research and Training, Guangdong Provincial Key Laboratory of Speed Capability Research, College of Chemistry and Materials Science, School of Physical Education, Jinan University, Guangzhou 510632, Chinayonghuanchen@stu2023.jnu.edu.cn (Y.C.);; 2 College of Chemistry, Zhengzhou University, Zhengzhou 450001, China

**Keywords:** cactus-inspired, osmosis-enrichment, deep learning, biosafety isolation, wearable sweat detection

## Abstract

Sweat, which contains a rich array of biomarkers, serves as a vital biological fluid for non-invasive biosensing. Wearable sweat sensors have garnered significant interest owing to their portability and capacity for continuous monitoring. However, there are safety concerns regarding the direct contact of sweat sensors with the skin during the detection process. The chemical substances in the sensor patches may cause contamination of the epidermis when in contact with the skin, leading to skin allergic reactions. Sample collection and biosafety isolation are critical issues in wearable sweat detection. To address this, we develop a cactus-inspired biomimetic Janus membrane capable of unidirectionally transporting and concentrating sweat toward a designated detection zone. Through unidirectional transport from the hydrophobic layer to the hydrophilic layer of the Janus membrane, sweat droplets are enriched at the designated detection point of the conical hydrophilic pattern via Laplace pressure. The bionic osmosis-enrichment sensing patch effectively inhibits direct contact between indicators and skin, eliminating potential epidermal contamination. This achieved the effect of in situ perspiration collection under the premise of biosafety isolation. To rapidly and accurately analyze sweat biomarkers, we employ a deep learning (DL)-assisted fluorescence sensor for efficient and precise detection of biomarker concentrations. A dataset of 4500 fluorescence images are constructed and used to evaluate two DL and seven machine learning (ML) algorithms. The convolutional neural network (CNN) model could easily and accurately classify and quantitatively analyze the total concentration of the amino acid mixture, Ca^2+^ and Cl^−^, with 100% classification accuracy. The consistency between the detection results of actual sweat by the DL-assisted fluorescence method and fluorescence spectroscopy was 91.4–96.0%. This approach demonstrates high reliability in sweat collection and analysis, offering a practical tool for clinical health monitoring, early disease intervention, and diagnosis.

## 1. Background and Originality Content

Personal health monitoring plays an increasingly crucial role in daily healthcare and chronic disease management [1]. Various methods have been explored to acquire vital signs from the human body [2,3,4]. As a representative biological fluid, sweat contains an abundance of biomarkers. Sweat analysis provides a simple, convenient, and non-invasive approach to obtain chemical constituents and biomarker information for daily health monitoring [5]. Serving as the fundamental building blocks of proteins and critical components in numerous metabolic processes, amino acids and their homeostatic balance within the organism are essential for maintaining physiological homeostasis and overall health [6]. Excessive fluctuations in ionized calcium levels in biological fluids can exert detrimental effects on the human body, including disorders such as acid–base imbalance and renal failure [7]. The chloride levels in sweat are critical for diagnosing and managing cystic fibrosis (CF), with clinical monitoring serving as a reliable tool for newborn screening, diagnosis, and treatment personalization [8]. The researchers developed methods for collecting and analyzing topical sweat in vitro, including sweat absorbing patches, textiles, and microfluidic channels [9,10,11]. However, there are safety concerns regarding the direct contact of sweat sensors with the skin during the detection process. The chemical substances in the sensor patches may cause contamination of the epidermis when in contact with the skin, leading to skin allergic reactions [12]. Sample collection and biosafety isolation are critical issues in wearable sweat detection. Therefore, there is a call for an efficient approach to rapidly collect sweat from the skin using non-contact or minimally adhesive wearable sensing devices or patches.

Janus membranes possess asymmetrical morphological structures or chemical compositions on either side, making them widely applicable in fields such as membrane distillation, oil–water separation, and seawater desalination [13,14,15]. The asymmetric wettability enables the unidirectional transport of droplets from the hydrophobic layer to the hydrophilic layer. The unidirectional permeability of the Janus membrane has a wide application prospect in the field of sweat collection [16,17,18]. As a medium for non-invasive detection, sweat can serve as a source of human health information. The Janus membrane can achieve unidirectional collection of sweat while isolating the sweat sensor from the skin. Pengyu Xi et al. proposed a colorimetric sweat sampling and sensing system based on the Janus membrane, where the opposite wettability enables sweat to quickly transfer from the skin surface to the hydrophilic surface [19,20]. The unidirectional sweat absorption performance not only facilitates adequate sweat sampling but also inhibits the reflux of reagents from the detection patch to the skin, thereby eliminating potential epidermal contamination. In addition, although the sweat glands are distributed in all parts of the body, the sweat secretion rates are low on the arms or legs [21]. In certain scenarios, the volume of sweat may not meet monitoring requirements. The spines of the cactus have a conical structure, and the droplets move spontaneously towards the bottom under the action of Laplace pressure [22]. Inspired by the special structure of the cactus, a conical hydrophilic pattern can be fabricated on a hydrophobic substrate to create a patterned Janus membrane. This approach can address the challenges of limited sweat volume and the lack of directionality during sweat collection.

Common methods for sweat analysis include colorimetry [23,24], fluorescence spectroscopy [25,26], electrochemistry [27,28], and surface-enhanced Raman spectroscopy (SERS) [29,30,31]. However, the accuracy of these strategies depends heavily on the performance of the instrument and the technical proficiency of the operator. Inherent disadvantages, such as the need for expensive equipment, time-consuming procedures, complex sample pretreatment processes, and trained technicians, limit their widespread use. Visual fluorescence sensors have attracted much attention in sweat sensing because of their excellent selectivity and sensitivity. However, data processing and analysis of fluorescent images inevitably require increased processing time and professional personnel. Furthermore, image datasets exhibiting nonlinearity and high complexity pose challenges for analysis using traditional statistical methods. With the rapid development of artificial intelligence and the increasing demand for data analysis in chemical research, DL technologies are being increasingly applied in chemistry, including in drug design [32,33], materials science [34], and analytical chemistry [35,36]. Convolutional neural network (CNN) is a branch of DL algorithms, offering significant advantages in processing high-dimensional data and solving nonlinear problems. It is widely used in image processing and recognition tasks [37,38,39]. In fluorescence image processing, CNN models can rapidly analyze the entire image and construct multi-parameter nonlinear models capturing the detection environment. However, due to the complexity of their parameter networks, the internal workings of CNN models are often considered a “black box” [40]. Consequently, it is difficult to discern which features the networks prioritize or rely upon during operation. To solve this problem, the class activation mapping (CAM) algorithm, which can generate attention maps, is increasingly being applied to explain the reasoning behind CNN-based decision making and feature extraction [41]. CAM intuitively reveals the feature dependencies of DL models when processing chemical sensing information.

**Figure 1:**
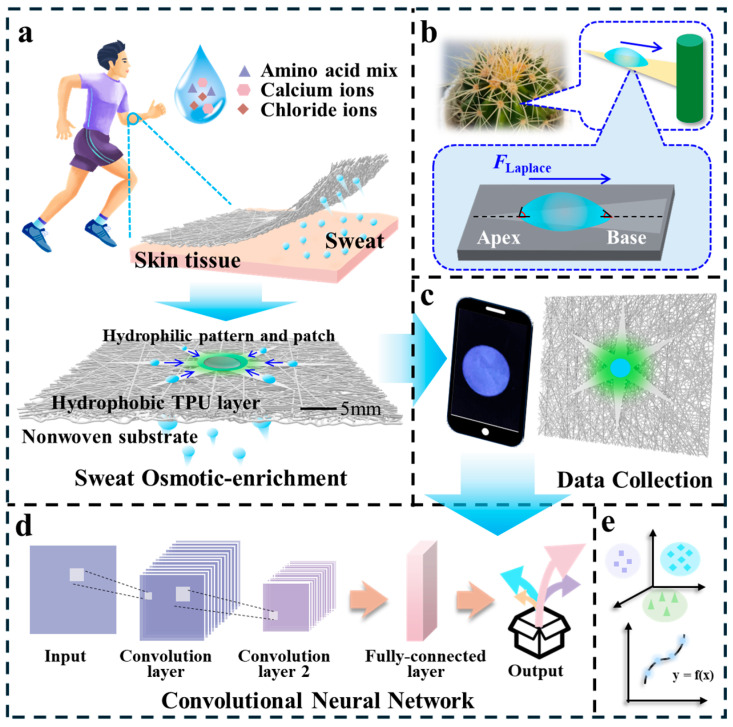
Schematic diagram of Janus membrane for spontaneous in situ sweat collection and DL-assisted programmable fluorescent microsensor patch for biomarker analysis. (**a**) Sweat is unidirectionally transported from the hydrophobic layer of the Janus membrane to the hydrophilic layer, and then from the hydrophilic layer’s conical tips to the designated location, where the hydrogel patch swells calibrated to the collected sweat volume and performs fluorescence analysis detection of the amino acid mixture, Ca^2+^, and Cl^−^ in the sweat. (**b**) Inspired by the conical spines of cacti and hydrophilic hairs, we designed conical hydrophilic patterns on a hydrophobic surface to manipulate droplets for continuous and effective unidirectional transport to designated locations. (**c**) Data collection through an intelligent photographic device. (**d**) The response information is input into an explainable CNN. (**e**) Classification and quantitative results of different biomarkers in the sweat are obtained.

In this study, we designed a cone-patterned Janus membrane for sweat collection, combining the functional advantages of fluorescence sensor and artificial intelligence algorithms for sweat collection and biomarker analysis. Inspired by the structure of the cactus, a conical hydrophilic pattern was fabricated on the hydrophobic, electrospun thermoplastic polyurethane (TPU) layer. After unidirectional transfer of droplets from the hydrophobic layer to the hydrophilic layer of the Janus membrane, the droplets were enriched to the center region of the conical hydrophilic pattern by the Laplacian force, as shown in Figure 1a. These biomarkers are essential for assessing an individual’s health status and guiding scientific training regimens. After collecting sweat, the fluorescence hydrogel sensor obtains information about the types and concentrations of amino acid mixture, Ca^2+^, and Cl^−^. By coupling with artificial intelligence algorithms to process and analyze the image data, the concentrations of amino acid mixture, Ca^2+^, and Cl^−^ in sweat can be readily classified and quantified, achieving 100% classification accuracy. The proposed Janus membrane and fluorescence sensor can reliably collect and analyze sweat. This work provides a strategy for the design and preparation of sensors capable of rapid sweat analysis, thereby offering a solution for personalized health monitoring and the early detection of developing health conditions.

## 2. Results and Discussion

### 2.1. Patterned Janus Membranes for In Situ Sweat Collection and Detection and DL-Assisted Fluorescence Sensor Chips for Sweat Analysis

Sweat contains information related to human health and metabolism. The analysis of its components and concentrations can provide an approach to non-invasive detection for health monitoring and instant diagnosis. However, the uneven distribution of sweat across the skin and its volatility pose significant challenges for in situ collection and detection. Inspired by the unique structure of cacti, a conical hydrophilic layer can be fabricated on a hydrophobic substrate to create a patterned Janus membrane. This design addresses the challenges of limited sweat volume and lack of directionality during collection. This membrane integrates binary interface wettability (hydrophilic layer–hydrophobic layer) and geometric asymmetric pattern hydrophilic layer structure, cleverly combining the dual liquid collecting mechanism of wettability gradient and Laplace pressure gradient to achieve efficient directional sweat collection (Figure 1a). Sweat can be unidirectionally transported from the hydrophobic layer to the hydrophilic layer under the action of the wettability gradient, and then transported from the planarized conical tip to the central area under the action of both gradients, providing conditions for in situ sweat detection (Figure 1b). For sweat component and concentration analysis, referencing previous work [42], we designed a programmable fluorescent hydrogel patch, prepared by crosslinking PVA and PAA. To satisfy the simultaneous detection of different components, various fluorescent indicators were selected to obtain fluorescent sensing information for the concentration analysis of sweat biomarkers. Specifically, we employed 2-mercaptoethanol-o-phthalaldehyde to detect the total amino acid mixture, Calcium Green™ for calcium ions, and Quinine Sulfate for chloride ions. More details about the indicators and data collection equipment are provided in the supporting materials. DL algorithms can perform a series of comprehensive nonlinear operations and have advantages in analyzing nonlinear and multidimensional data. After the collected fluorescent images are input into an explainable CNN (Figure 1d), the classification and quantitative results for each sweat component are obtained (Figure 1e). Additionally, the CAM was utilized to generate attention maps, elucidating the rationale behind CNN-based decisions in sweat biomarker analysis. These evaluation results serve as feedback, which validates the rationality of the patch design and fabrication process, guides the refinement of programmable fluorescence methods, minimizes algorithmic redundancy, streamlines operations, enables efficient data collection, and ultimately supports the development of flexible and reliable biomarker detection strategies.

The bio-inspired Janus membrane and fluorescent hydrogel sensing patch can be integrated onto a flexible substrate to form a complete wearable system. In practical applications, the patch can be attached to human skin, enabling directional sweat collection and enrichment through the Janus membrane. Upon contact with sweat, the fluorescent hydrogel patch exhibits a fluorescent response. Subsequently, images are captured by a portable imaging device and transmitted to a deep learning model deployed locally or in the cloud for analysis. Ultimately, the classification and concentration results of biomarkers are displayed in real-time on the user terminal.

### 2.2. Janus Membrane Structure and Morphology Characterization

**Figure 2:**
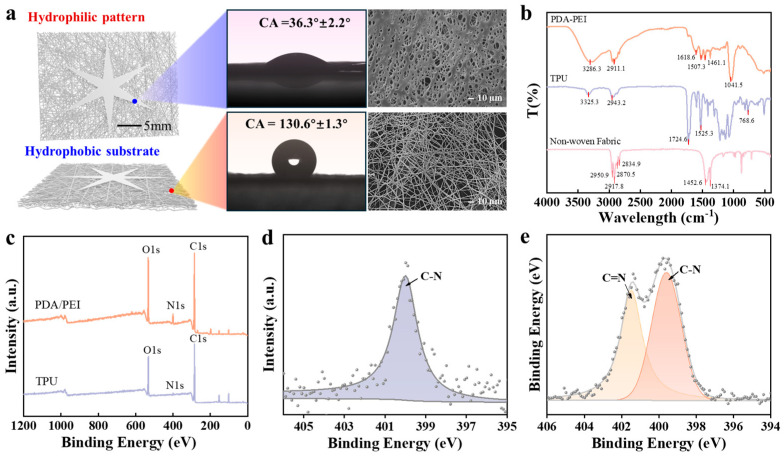
Morphology and structural characterization of the Janus membrane for spontaneous in situ sweat collection. (**a**) SEM images of the hydrophilic and hydrophobic regions of the Janus membrane and their corresponding static contact angles. (**b**) FT-IR spectra of the three layers of the Janus membrane. (**c**) XPS spectra and N1s fine spectra of the hydrophobic and hydrophilic layers, (**d**) TPU and (**e**) PDA/PEI, respectively.

The Janus membrane consists of three layers, as illustrated in Figure 2a. The first layer is a non-woven fabric support layer, primarily composed of polypropylene; its morphology and fiber diameter are shown in Figure S1. The second layer is a hydrophobic TPU layer, fabricated via electrospinning for 50 min, exhibiting a contact angle of 130.6° ± 1.3° and a fibrous morphology with an average fiber diameter of 1.71 ± 0.49 μm. The density of fibers in the hydrophobic layer is related to the duration of electrospinning (Figure S2). The third layer, a patterned multi-conical hydrophilic layer, is formed by depositing PDA/PEI onto the TPU fibers, displaying a porous structure with a contact angle of 36.3° ± 2.2°. The variation in the contact angle with deposition time and PEI concentration is shown in Figure S3a. In the FT-IR spectrum (Figure 2b), the characteristic peaks of polypropylene observed at 2917.8 and 2870.5 cm^−1^ correspond to the stretching vibrations of -CH_3_. The characteristic peaks at 1374.1 and 1452.6 cm^−1^ are attributed to the bending vibrations of -CH_3_ and -CH_2_-. For TPU, the characteristic peaks at 3325.3 and 2943.2 cm^−1^ are mainly due to the stretching and bending vibrations of -NH- and -CH- in the polyurethane. The vibrational peaks at 1724.6 and 1525.3 cm^−1^ belong to the -NH-COO- group in the polyurethane. After the deposition of PDA/PEI on the TPU layer, the absorption peaks at 1618.6 and 1507.3 cm^−1^ correspond to the C=C resonance vibration from aromatic rings and the bending vibration of -N-H-, indicating the presence of PDA, and the characteristic peak at 3286.3 cm^−1^ corresponds to the stretching vibration of -NH-. X-ray photoelectron spectroscopy (XPS) is employed to characterize the chemical composition of the prepared samples (Figure 2c). Both the TPU and PDA/PEI layers contain C, N, and O elements, with an increase in the peak intensity of N1s in the PDA/PEI layer, where the nitrogen content increased from 1.32 to 6.17%. In Figure 2e, the fine spectrum of N1s in PDA/PEI shows peaks at 399.59 and 401.48 eV, corresponding to C-N and C=N bonds, respectively. Compared to the peak at 400.0 eV attributed to C-N in the TPU spectrum (Figure 2d), this shift indicates that PEI undergoes Michael addition or Schiff base reactions between amine groups and catechol moieties, forming C=N bonds.

### 2.3. Spontaneous Droplet Sampling

**Figure 3:**
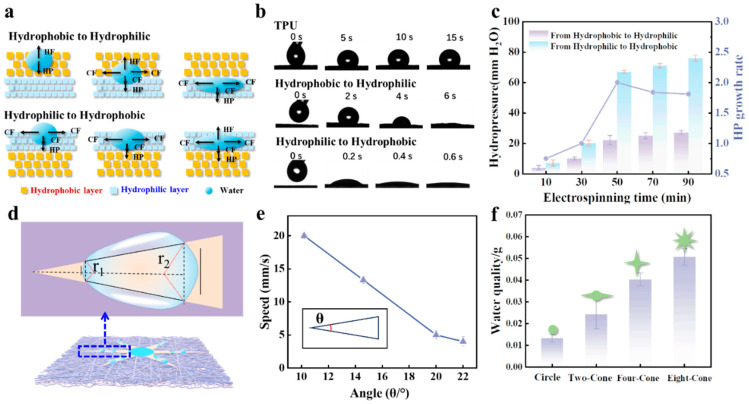
Unidirectional transport and enrichment of sweat. (**a**) Schematic diagram of the unidirectional transport mechanism of the Janus membrane (HP: Hydrostatic Pressure; HF: Hydrophobic Force; CF: Capillary Force). (**b**) Continuous WCA of the Janus membrane. (**c**) The relationship between HP on both sides of the Janus membrane and the electrospinning time used for the TPU fiber membrane. (**d**) Schematic diagram of unidirectional droplet transportation caused by the Laplace pressure gradient. (**e**) Droplet movement speed on hydrophilic layers with different conical pattern angles. (**f**) Droplet collection capacity in grams for Janus membranes with different numbers of cones on the hydrophilic layer. Error bars represent the standard deviations from three independent experiments (n = 3).

The unique wettability of the Janus membrane results in unidirectional water transport behavior, as shown in Figure S3, where water droplets penetrate from the hydrophobic layer to the hydrophilic layer. However, when the Janus membrane is inverted, water droplets are blocked and spread solely on the hydrophilic layer without penetrating the membrane. The mechanism underlying the unidirectional water transport of the Janus membrane is illustrated in Figure 3a. When the water droplet resides on the hydrophobic layer, it initially exists in a Wenzel–Cassie state and experiences two opposing forces: the hydrophobic force (HF) and hydrostatic pressure (HP). HF relates to the break-through pressure of the hydrophobic layer, which prevents water penetration. For a given membrane, HF is constant. Conversely, HP is proportional to the height of the water column, enabling water to pass through vertical porous channels and penetrate the membrane. Once HP exceeds HF, the droplet fully wets the rough surface, transitioning to a Wenzel state. In practice, the thicker the hydrophobic layer, the greater the HP required for complete penetration. Once water penetrates the hydrophobic layer into the hydrophilic layer, both CF (capillary force) and HP jointly facilitate its diffusion and penetration until it completely traverses the Janus membrane.

Conversely, when the water droplet is placed on the hydrophilic layer, the CF provided by the PDA/PEI hydrophilic layer causes the water to spread on the surface. When the droplet reaches the interface between the hydrophilic and hydrophobic layers, the HF provided by the hydrophobic layer blocks further penetration. Increasing the volume of water on the hydrophilic layer enlarges the diffusion area but does not significantly increase the water height. Consequently, HP remains lower than HF, preventing water penetration into the hydrophobic layer. Similar to droplets on cactus conical spines and hydrophilic hairs, the droplet reaching the hydrophilic layer experiences Laplace pressure generated by the gradient wettability and conical patterns, driving its unidirectional transport. Figure 3b presents the evolution of the water contact angle (WCA) over time for TPU and Janus membranes. The TPU fiber membrane exhibits strong hydrophobicity, as evidenced by the minimal change in the water droplet’s shape over time. The droplet on the TPU layer of the Janus membrane penetrates to the hydrophilic layer within 6 s and spreads rapidly on the PDA/PEI layer (Figure S4), demonstrating excellent unidirectional permeability from the hydrophobic side to the hydrophilic side. To further investigate the influence of water on both sides of the Janus membrane, the HP required to pass water through the porous channels of the membrane was measured. Using a fixed area of the Janus membrane, water was slowly dripped from a tubular container. The height of the water column at the onset of penetration was recorded, allowing measurement of HP values for transport from the hydrophobic (TPU) side to the hydrophilic (PDA/PEI) side and vice versa. Figure 3c shows the relationship between TPU electrospinning time and HP. The HP required for transport from the hydrophobic (TPU) side to the hydrophilic layer is significantly lower than that for the reverse direction, confirming that water droplets permeate more readily from the hydrophobic to the hydrophilic side.

As the TPU electrospinning time increases, the corresponding HP increases due to the longer hydrophobic channels that the water must traverse, making water transport more difficult. By comparing the rate of increase in HP, an electrospinning time of 50 min was selected for the hydrophobic layer. Inspired by the conical structure of cacti, an asymmetrically structured, conical-patterned hydrophilic layer was fabricated on a hydrophobic substrate. The pattern of the hydrophilic layer gradually expands from the top of the cone to the bottom. When a droplet is transported to the conical pattern hydrophilic layer, it undergoes asymmetric deformation confined within the conical channels, forming droplets with different curvature radii. This is manifested by the difference in the radius of curvature (r_1_ and r_2_) at the three-phase contact line (TCL) between the front and back of the droplet (Figure 3d). The curvature radius near the tip is smaller, while the curvature radius near the base is relatively larger. Due to r_1_ < r_2_, an imbalanced surface tension generates Laplace pressure (ΔP) from the side with a smaller curvature to the side with a larger curvature:

(1) $$\Delta P=\gamma \left(\frac{1}{r_{1}}-\frac{1}{r_{2}}\right)$$

where γ is the surface tension of the droplet.

For the conical patterned hydrophilic layer, the droplet confined in the conical channel undergo asymmetric deformation, showing different curvature radii in front and back. The unbalanced surface tension generates Laplace pressure ΔP from the side with smaller curvature to the side with larger curvature, causing the droplet to move from the tip of the cone to the base. In this case, when the droplet penetrates from the hydrophobic surface of the Janus membrane to the patterned hydrophilic surface, the droplet will spontaneously move towards the base of the conical pattern. The droplet’s movement speed is related to the angle θ of the cone tip. The larger the angle, the smaller the Laplace pressure difference, and the slower the droplet moves. Figure 3e compares the movement speeds for conical pattern tips with angles of 10.2°, 14.6°, 20°, and 22°. When the conical pattern angle is 10.2°, the movement speed can reach 20 mm/s. The dimensions and surface area of the hydrophilic patterns constitute another critical set of parameters governing droplet transport and enrichment efficiency. While the cone angle (θ) directly determines the Laplace pressure gradient, the absolute dimensions of the patterns define the effective surface area available for droplet wetting and the total liquid capacity. In this work, based on circular hydrophilic spots (with a diameter of 7 mm), we fabricated hydrophilic layers featuring different numbers of conical patterns—such as double, quadruple, and octuple cones, all with a vertex angle of 10.2° and a height of 10 mm (Figure S5). This design strategy systematically increases the total active surface area for liquid convergence, thereby directly enhancing the enrichment capability. Quantitative results in Figure 3f demonstrate that the Janus membrane with an octuple-cone pattern exhibits a 3.85-fold improvement in droplet collection capacity compared to a membrane with a single circular pattern. These findings indicate that, within a certain range, a greater number of conical tips and a larger surface area of the hydrophilic patterns lead to more efficient sweat collection under low-secretion-rate conditions.

### 2.4. The Chemical Sensing Mechanism of Sweat Biomarkers

Due to the complexity of human sweat composition and the variability in sweat volume, quantitative detection of biomarkers in sweat has always been a challenge. To overcome this difficulty, we adopted an approach that combines programmable fluorescent chips with explainable DL algorithms for biomarker detection. In this study, the concentration ranges of selected sweat biomarkers were as follows: amino acid mixture 1 × 10^−5^ to 9 × 10^−3^ mol/L, Ca^2+^ 1 × 10^−4^ to 9 × 10^−2^ mol/L, and Cl^−^ 1 × 10^−4^ to 9 × 10^−2^ mol/L. The amino acid mixture comprised eight distinct components with the following concentrations: serine 0.4167 g/L, glycine 0.2083 g/L, glutamate 0.1389 g/L, aspartate 0.0694 g/L, threonine 0.0694 g/L, histidine 0.0694 g/L, leucine 0.0417 g/L, and valine 0.0417 g/L.

**Figure 4:**
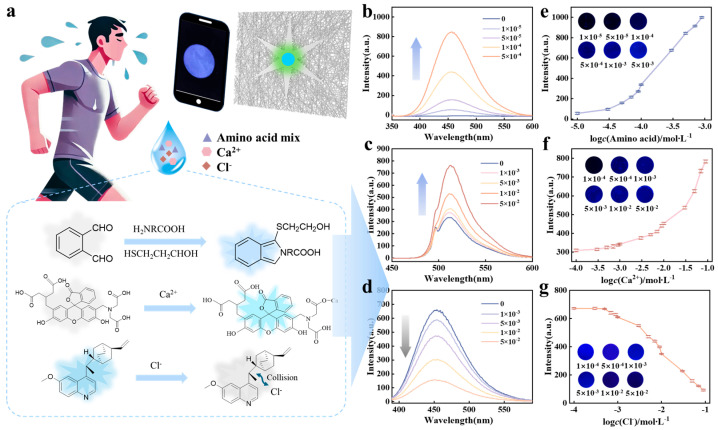
Chemical sensing mechanism of the fluorescent hydrogel patch and corresponding spectra. (**a**) The chemical fluorescence response mechanism of indicators to sweat biomarkers. (**b**–**d**) Corresponding spectra for the response of selected indicators to different sweat biomarkers. (**e**) Optical images and fluorescence intensity plots for amino acid mixture (1 × 10^−5^ to 9 × 10^−3^ mol/L), (**f**) Ca^2+^ (1 × 10^−4^ to 9 × 10^−2^ mol/L), and (**g**) Cl^−^ (1 × 10^−4^ to 9 × 10^−2^ mol/L). Error bars represent the standard deviation from three independent experiments (n = 3).

The chemical sensing mechanisms of the fluorescent hydrogel patch for sweat biomarkers and the corresponding spectra are presented in Figure 4. When 2-mercaptoethanol serves as a reducing agent, o-phthalaldehyde reacts with amino acid mixture via cyclization and condensation reactions, forming highly fluorescent derivatives. Upon excitation at 347 nm, the fluorescence intensity is directly proportional to amino acid mixture concentration, as shown in Figure 4b. The detection mechanism for Ca^2+^ relies on the fluorescence enhancement upon binding of Calcium Green™ to calcium ions (Figure 4c). For Cl^−^ detection, quinine sulfate is chosen as a fluorescent indicator, which exhibits intense blue emission fluorescence that quenched in the presence of chloride ions (Figure 4d). Figure 4e–g show the optical images and corresponding fluorescence spectral intensities of these sweat biomarkers at different concentration levels across various concentration gradients. The fluorescence intensity for each biomarker exhibits a linear relationship with concentration only within a specific range.

### 2.5. DL-Assisted Detection of Sweat Biomarkers Using Fluorescence Chips

**Figure 5:**
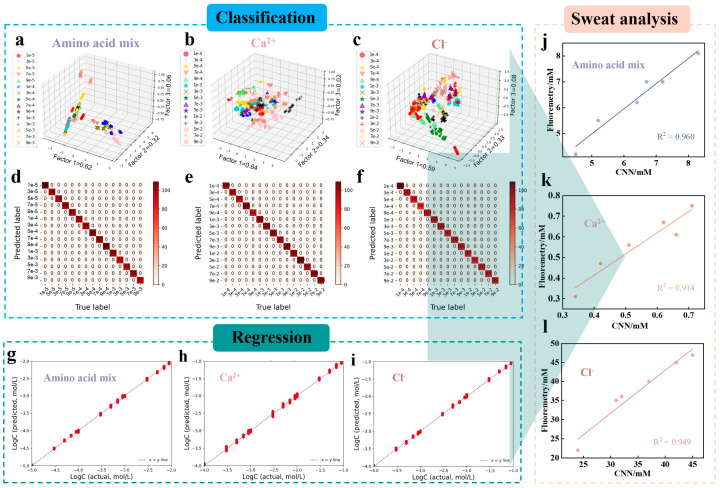
Classification and quantitative analysis of various sweat biomarkers using ML and DL models. (**a**) Three-dimensional LDA score plots for amino acid mixture (1 × 10^−5^ to 9 × 10^−3^ mol/L), (**b**) Ca^2+^ (1 × 10^−4^ to 9 × 10^−2^ mol/L), and (**c**) Cl^−^ (1 × 10^−4^ to 9 × 10^−2^ mol/L). Confusion matrices for CNN predictions of (**d**) amino acid mixture, (**e**) Ca^2+^, and (**f**) Cl^−^. (**g**–**i**) Match between actual and predicted concentrations in the test set for CNN-based quantitative analysis. (**j**–**l**) Accuracy assessment comparing the prediction results from CNN-assisted fluorescent patches with those from fluorescence spectroscopy measurements.

In this work, we analyzed fifteen concentration levels for each of three biomarker categories: amino acid mixture (1 × 10^−5^ to 9 × 10^−3^ mol/L), Ca^2+^ (1 × 10^−4^ to 9 × 10^−2^ mol/L), and Cl^−^ (1 × 10^−4^ to 9 × 10^−2^ mol/L). For each concentration, ten independent replicate experiments were performed. Each replicate hydrogel patch was imaged at ten different positions to account for spatial variations, yielding a total of 3 × 15 × 10 × 10 = 4500 fluorescence images of the hydrogel patches. This comprehensive set of images constitutes the complete dataset used for subsequent analysis. The dataset was randomly partitioned into training, validation, and test sets at an 8:1:1 ratio. The training set was used for model training, the validation set for adjusting hyperparameters and preliminarily evaluating model capabilities, and the test set for assessing the final model’s generalization ability. In machine learning (ML), linear discriminant analysis (LDA) results for classifying sweat biomarkers are shown in Figure 5a–c; it failed to completely distinguish between different concentrations of biomarkers. There is significant cluster overlap, with classification accuracies of 60.7–62.8% for amino acid mixture, 33.3–46.3% for Ca^2+^, and 25.9–27.9% for Cl^−^. These results indicate that the evaluated ML models lack sufficient sensitivity for multivariate fluorescence analysis.

Subsequently, advanced analysis was conducted using DL algorithms. The ResNet-18 network, known for its CNN residual learning framework, can optimize the network and achieve accuracy by progressively increasing depth. We designed a reasonable CNN structure based on the ResNet-18 network and optimized the model’s hyperparameters, including the number of epochs, batch size, and learning rate (see Supplementary Table S1). The CNN model effectively extracts features from the fluorescence response data, enabling accurate classification as evidenced by the confusion matrices (Figure 5d–f). As shown in Supplementary Table S2, the selected three ions/molecules achieved 100% accuracy across all three subsets, demonstrating that the CNN model’s classification accuracy surpasses that of the LDA model.

We investigated two DL or seven ML models, including CNN, ANN, XGBoost, DT, KNN, logistic regression (LR), naive Bayes (NB), random forests (RF), and support vector machines (SVM) (model structures and parameters are detailed in Supplementary Tables S3–S4). While ANN, XGBoost, KNN, RF, and DT models showed good fitting capability in the amino acid mixture classification task and nonlinear description, their classification accuracies for Ca^2+^ and Cl^−^ on both the validation and test sets decreased to varying degrees. Only the CNN model displayed 100% accuracy across all three subsets (Figure S6 and Supplementary Table S2). This is due to the following reasons: (1) DL models often have complex structures that allow them to handle more intricate nonlinear relationships, leading to better performance in classification tasks. Additionally, by tuning the model’s hyperparameters, further optimization of the model’s performance can be achieved. (2) DL models can automatically extract high-level feature representations from raw data, which are often more discriminative and effective than handcrafted features derived from traditional ML methods.

**Figure 6:**
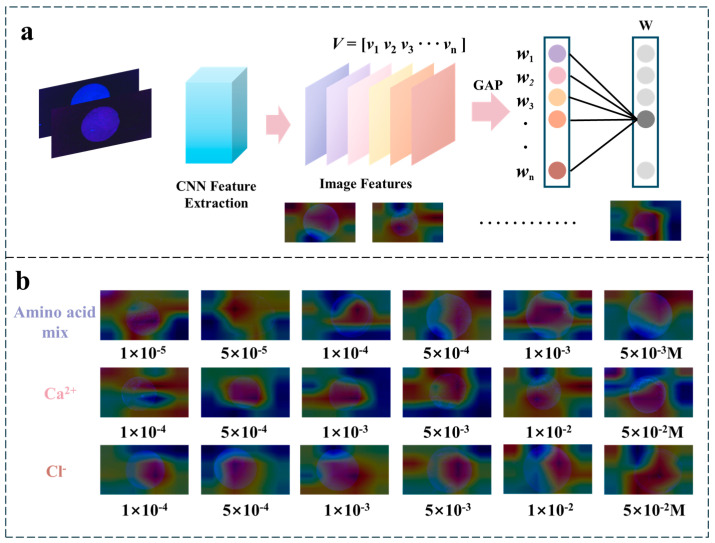
CNN explains the mechanism of CAM sensing patch. (**a**) Feature extraction based on CAM mechanism: provide “interpretation” for the results of the CNN model, open the “black box” of the CNN model, and mine the most critical chemical sensing information behind the decision-making mechanism of the CNN model. (**b**) CAM maps of selected sweat biomarkers (amino acid mixture, Ca^2+^, and Cl^−^) based on CNN model results.

Figure 4e–g present optical images of these sweat biomarkers at different concentration levels along with their corresponding fluorescence spectral responses. Figure 6a introduce a CNN quantification model we developed (for specific architecture, see Supplementary Table S5). This model aims to precisely predict the specific concentration values of the studied biomarkers. With this technology, our DL-assisted programmable fluorescence patch can accurately assess the concentrations of amino acid mixture, Ca^2+^, and Cl^−^ in actual samples and predicted samples within the specified range. To verify the accuracy of the established model, we employed various quantitative performance metrics for evaluation (as shown in Supplementary Tables S6–S8), including the coefficient of determination (R^2^), mean squared error (MSE), root mean squared error (RMSE), and mean absolute error (MAE). Scatter plot analysis of the relationship between predicted results and true values on the test set revealed that the model performed excellently, with R^2^ values exceeding 0.999. Additionally, the linear fit slope between predicted and actual values was very close to the ideal state of 1, indicating a high degree of consistency between them. Moreover, when comparing CNN with several other common DL/ML algorithms such as ANN, XGBoost, DT, KNN, LR, RF, and SVM. Although some methods like ANN, XGBoost, and RF achieved good performance in amino acid mixture detection modeling, they exhibited varying degrees of overfitting issues during subsequent modeling of the other two ions, failing to demonstrate good generalization ability. In contrast, CNN not only significantly reduced the possibility of overfitting but also exhibited superior generalization performance (more details can be found in Supplementary Tables S6–S10).

Individual differences can affect sweat biomarkers. Leveraging the excellent quantitative analysis capability of the DL-assisted fluorescent patch, we further investigated the concentration levels of sweat biomarkers in six subjects under exercise conditions. The DL predictions for different subjects’ biomarker concentrations are shown in Figures S7a–c. The total concentration of amino acid mixture in human sweat is about 10^−3^ mol/L, Ca^2+^ concentration is about 10^−4^ to 10^−3^ mol/L, and Cl^−^ concentration is about 10^−2^ mol/L. These results are consistent with the reported normal physiological range for humans. To validate the accuracy of the DL-assisted programmable patch, we compared it with laboratory fluorescence spectroscopy (Figure 5j–l). We found that the test results from the DL-assisted fluorescent patch matched the laboratory measurements with a rate of 91.4−96.0%. The limits of detection for the amino acid mixture, Ca^2+^, and Cl^−^ are 1.0 × 10^−5^, 6.7 × 10^−5^, and 1.0 × 10^−4^ mol/L, respectively. The above results effectively demonstrate the reliable analysis and practical applicability of the DL-assisted programmable fluorescence patch in quickly and accurately evaluating sweat.

To evaluate the reliability of the sensing platform for practical sweat detection, we further investigated the selectivity of the fluorescent hydrogel patches against common interferents and their long-term stability. As shown in Figure S8, the fluorescence responses elicited by the target analytes (amino acid mixture, Ca^2+^, Cl^−^) were significantly stronger than those from potential interferents in sweat (such as Na^+^, K^+^, glucose, lactate, and urea) at physiologically relevant concentrations, demonstrating excellent selectivity of the sensor. Furthermore, the hydrogel patches were subjected to a ten-day stability test. The results (Figure S9) confirmed no significant variation in their detection performance, underscoring the robust stability of the sensor for practical applications.

### 2.6. The Interpretability of CNN for Fluorescent Patch

During CNN model training, the internal architecture extracts feature information from images through successive convolutional operations. This process generates highly abstract representations, which makes the internal decision-making process of the DL model challenging to fully comprehend. CAM technology can unveil the internal mechanisms of the network, making the sensing process visualized. Figure 6a shows a schematic diagram of the CAM, illustrating feature extraction for response data classification. Each neuron is a linear combination of neurons from the previous layer, obtained through a series of nonlinear functions. Figure 6b presents the CAM heatmap, which visualizes the attention mechanism of the CNN during classification. Warmer colors (red) indicate higher network attention, while cooler colors (blue) indicate lower attention. Analysis of the CAM maps for the amino acid mixture, Ca^2+^, and Cl^−^ leads to the following mechanistic insights: (1) The CNN primarily focuses on regions exhibiting fluorescence intensity changes within the sensing patch, utilizing this information end-to-end without additional human intervention. (2) The regions of high CAM activity (red areas) align with the chemical sensing design of the fluorescent patch. The designed fluorescent hydrogel sensing patch provides the requisite features for DL analysis, and the interpretability of the DL model can be validated by testing the performance of the network model to eliminate potential errors or risks. The insights gained from CAM can guide software design, inform feature extraction strategies, and refine theoretical models. This approach provides convenient indicators for health management, disease prevention, and even new scientific discoveries.

## 3. Conclusions

In summary, we present a wearable, cactus-inspired patterned Janus membrane integrated with an explainable deep learning (DL)-assisted fluorescent hydrogel sensing platform for the spontaneous collection and efficient, accurate detection of sweat biomarkers. The patterned Janus membrane is fabricated by fabricating a conical patterned hydrophilic layer on the electrospun TPU. Leveraging hydrostatic pressure and Laplace pressure, this design enables a dual process: unidirectional sweat transport from the hydrophobic to the hydrophilic layer, followed by unidirectional movement and enrichment within the conical hydrophilic pattern. Capitalizing on the advantages of explainable DL algorithms, we develop a CNN-assisted PVA-PAA fluorescent hydrogel sensor with immobilized fluorescent indicators. An analytical dataset comprising 4500 fluorescence images is collected, and two DL algorithms along with seven ML algorithms are evaluated. The CNN model can accurately classify and quantify the concentration of the amino acid mixture, Ca^2+^ and Cl^−^ in sweat, achieving 100% accurate classification and 99.9% quantitative prediction over a wide concentration range. Results obtained from actual sweat using the DL-assisted fluorescence sensor show 91.4–96.0% agreement with conventional laboratory methods, providing a simple and convenient approach for rapid detection, disease intervention and clinical diagnosis. The cactus-inspired sweat osmosis-enrichment patch can achieve the effect of in situ perspiration collection under the premise of biosafety isolation for long-term wearable sweat metabolism monitoring.

## 4. Experimental

### 4.1. Fabrication of the Patterned Janus Membrane

The hydrophobic layer TPU (15%) was spun on the non-woven fabric by electrospinning technology. The spinning solution was prepared by dissolving 1.8 g TPU in 5 mL DMF and 5 mL THF. Then the mixed spinning solution was transferred to a syringe (10 mL). In the experiment, a high-voltage power supply was used to apply a voltage of 15 kV. The diameter of the syringe needle was 0.8 mm and the injection rate was 0.08 mL/h. The TPU was received on a rotary receiver fixed with non-woven fabric, and the receiver was 10 cm away from the syringe. The hydrophobic TPU was obtained after spinning for a certain time.

Subsequently, molds featuring different numbers of cones were fabricated via 3D printing. A thin layer of polydimethylsiloxane was applied to the molds and then heated in an oven at 70 °C for 3 h to obtain hydrophobic molds. Molds with varying hydrophobic patterns were fixed on the TPU hydrophobic layer. A certain volume of dopamine hydrochloride and polyethyleneimine (DA/PEI) solution was added to the molds. After 12 h, patterned Janus membranes with circular, double-cone, quadruple-cone, and octuple-cone hydrophilic patterns were obtained (Figure S10).

### 4.2. Preparation of Programmable Fluorescent Hydrogel Chips

Fluorescent indicators (2-mercaptoethanol-o-phthalaldehyde for amino acid mixture, Calcium Green™ for Ca^2+^, Quinine Sulfate for Cl^−^) were prepared and solidified into PVA-PAA fluorescent hydrogel patches for detecting biomarkers in sweat. A certain amount of fluorescent indicators, 1.000 g PAA, and 4.000 g PVA were dissolved in 100 mL of deionized water. The solution was stirred and dissolved at 100 °C in an oil bath pot and then left to stand for 12 h to remove air bubbles, resulting in a fluorescent indicator-fixed PVA-PAA hydrogel precursor solution (Figure S11). The hydrogel precursor solution was poured into surface plates with a depth of 5 mm and dried using a dehumidifier for 12 h to obtain fluorescent hydrogel films with a thickness of 0.10 ± 0.03 mm. Finally, a hole punch with a diameter of 5 mm was used to cut out several patches with a direct size of 5.0 ± 0.03 mm for detecting biomarkers in sweat.

## Data Availability

Data will be made available on request.

## Supplementary Materials

The following supporting information can be downloaded at: https://www.mdpi.com/article/10.3390/bios15120790/s1. Figure S1: SEM and particle size distribution of 10 g/m^2^ non-woven fabric (a) and 50 min TPU electrospinning (b); Figure S2: SEM images of different electrospinning time on non-woven fabric; Figure S3: The change of contact Angle of the three layers after deposition of different PEI concentrations (a), the time for 5 µL water droplets to reach the surface of the nonharmful fabric layer until the contact Angle decreases to zero (b), and the time-displacement diagram (c) and velocity diagram (d) for 10 µL water droplets to move from the conical tip to the wide end; Figure S4: Water droplets are transported from the hydrophilic layer (a) and the hydrophobic layer (b); Figure S5: (a) Schematic drawings of patterned molds for circles and different numbers of cones. (b) Schematic drawings of patterned Janus membranes with circles and different numbers of cones; Figure S6: Different classification models (including ANN, XGBoost, DT, KNN, LR, NB, RF, SVM and CNN) were used to compare the prediction accuracy of sweat biomarkers (amino acid mixture, Ca^2+^, and Cl^−^) on the training set, validation set and testing set; Figure S7: Quantitative determination of amino acid mixture, Ca^2+^ and Cl^−^ in actual sweat of six subjects during exercise. (a-c) Prediction of amino acid mixture, Ca^2+^, and Cl^−^ concentrations by DL-assisted programmable self-calibrating fluorescent patches. (d-f) Evaluation of the accuracy of prediction results of CNN-assisted fluorescent patches compared to fluorescence spectral detection results; Figure S8: Selectivity of (a) amino acid mixture, (b) Cl^−^, and (c) Ca^2+^ in programmable fluorescent hydrogel chips; Figure S9: Stability of (a) amino acid mixture, (b) Ca^2+^, and (c) Cl^−^ in programmable fluorescent hydrogel chips; Figure S10: Spontaneous in situ sweat collection patterned Janus membrane preparation; Figure S11: Preparation process of wearable programmable fluorescent hydrogel patch; Figure S12: Diagram of 5 µL water droplets moving from the hydrophobic and hydrophilic boundaries of the patterned Janus membrane with circles and different numbers of cones; Figure S13: Water droplets are transported from the conical tip and wide end; Table S1: The architecture of the CNN classification model; Table S2: Classification results of DL and ML methods; Table S3: The architecture of the ANN classification model; Table S4: Parameters of ML classification models; Table S5: The architecture of the CNN quantification model; Table S6: Quantitation results of DL and ML methods (Amino acid mixture); Table S7: Quantitation results of DL and ML methods (Cl^−^); Table S8: Quantitation results of DL and ML methods (Ca^2+^); Table S9: The architecture of the ANN quantification model; Table S10: Parameters of ML quantification models.
