# Supplementary material for: Deep Learning-Assisted Cactus-Inspired Osmosis-Enrichment Patch for Biosafety-Isolative Wearable Sweat Metabolism Assessment
Source: Biosensors (Basel). 2025 Dec 1;15(12):790. doi: 10.3390/bios15120790 (PMC12730226; doi:10.3390/bios15120790)
Supplement: Supplementary file 1 [file biosensors-15-00790-s001.zip › biosensors-3938283-supplementary.pdf]

Supporting Information

Experimental section

1.Chemicals and reagents

All chemicals are commercially available and have not been further purified. Among them, NaCl, $CaCl_2$, quinine sulfate were purchased from Tianjin Damao Chemical Reagent Factory. Calcein and 2-mercaptoethanol-o-phthalaldehyde were purchased from Sinopsin Chemical Reagent Co., Ltd. β-mercaptoethanol, serine, glutamic acid, glycine, aspartic acid, threonine, histidine, and leucine were purchased from Shanghai Maclin Biochemical Technology Co., td. Sulfuric acid was purchased from Guangzhou Chemical Reagent Factory.

2.Instruments and devices

The Frontier Fourier Infrared Spectrometer was purchased from PerlinElmer and the MIRA 3 LMU Field Emission Scanning Electron Microscope (SEM) was purchased from TESCAN Brno s.r.o. (Czech) Co., Ltd. BSA124S analytical balance was purchased from Sartorius Scientific Instruments (Beijing) Co., Ltd. SHL-500 smart camera was purchased from Shenzhen Shunhuali Electronics Co., Ltd. RF-5301 fluorescence spectrophotometer was purchased from Shimadzu Corporation. PHS-3C precision pH meter was purchased from Shanghai Precision Scientific Instrument Co., Ltd.

3.Preparation of fluorescent hydrogel patch

Fluorescent indicators (including 2-mercaptoethanol-o-phthalaldehyde, Calcium Green^{TM}, Qui-nine Sulfate) were prepared and solidified into PVA-PAA fluorescent hydrogel patches for detecting biomarkers in sweat. A certain amount of fluorescent indicators, 1.000 g PAA, and 4.000 g PVA were added to 100 mL of deionized water. The solution was stirred and dissolved at 100 °C in an oil bath pot and then left to stand for 12 hours to remove bubbles, resulting in a fluorescent indicator-fixed PVA-PAA hydrogel solution. The hydrogel solution was poured into surface plat. Then, we ventilated and dried the gel solution into a surface plate with a control liquid depth of 5 mm and dried it at room temperature for 12 hours to obtain a film with a thickness of 0.10 ± 0.03 mm. Finally, the membrane was cut into indicator immobilized PVA-PAA colorimetric patch with a diameter of 5 ± 0.03 mm using a hole punch.

4.Hardware and software configuration

The program is written in python language, wherein PIL library processes image data sets, OpenCV library reads pixel values of data sets, and Numpy library processes pixel values into matrices. All Code is run in Microsoft Windows 10 (x64) Visual Studio Code(version 1.76.2), the computer's CPU version is Intel Core i5-5200u, and the memory is 12GB. All DL algorithms (CNN, ANN model) are built by Pytorch, and

all ML algorithms are built by scikit-learn library. The XGBoost model is built from the XGBoost library.

5.Data collection and analysis

To ensure statistical robustness and reliable evaluation of the method's repeatability, the following experimental design was implemented: In our work, we tested 15 concentration levels for each of the three categories of biomarkers (amino acid mixture, Ca^{2+}, and Cl^{-}). At each concentration level, ten independent parallel samples (n=10) were prepared. To account for potential spatial heterogeneity in the fluorescence signal and to augment the dataset for the subsequent deep learning analysis, each independent parallel sample was imaged 10 times at different positions within the sensor, generating 10 images per replicate. Therefore, the total number of images collected was calculated as follows: 3 biomarkers × 15 concentrations × 10 parallel samples × 10 images per sample = 4,500 images. The data from the ten parallel samples (n=10) at each concentration formed the basis for assessing the reproducibility of the fluorescent hydrogel patches themselves. To account for potential spatial heterogeneity and augment the dataset for robust deep learning, each hydrogel patch (physical replicate) was imaged at ten distinct locations, generating ten sub-sampled images per replicate. Subsequently, the larger image dataset (100 images per concentration, derived from the 10 sub-sampled images × 10 images each) was utilized for training and validating the deep learning models, ensuring that the models learned robust and generalizable features rather than memorizing specific imaging artifacts. Those optical images were obtained with the SHL-500 electronic camera and S-EYE photography software. The shooting parameters of the SHL-500 electronic camera are set to: resolution and format of 1920×1080 jpg, exposure time of 0, gain of 10, color temperature of 6500, brightness of 0, contrast of 0, saturation of 177, hue of 0, gamma of 1, sharpness of 1. The obtained non-repetitive fluorescence images were used as a dataset with 100 images each for each concentration and each volume. The entire data set is divided into three subsets: the training set, the validation set, and the external unlabeled test set, with a ratio of 8:1:1.

6.Classification model

The program is written in Python and uses two deep learning algorithms: Convolutional Neural Network (CNN) and Artificial Natural Network (ANN) models and eight machine learning algorithms Naive Bayes (NB), Principal Component Analysis (PCA), Logistic Regression (LR), Decision Tree (DT), Random Forest (RF), Support Vector Machine (SVM), K-Nearest Neighbors (KNN), Extreme Gradient Boosting (XGBoost) to build a classification model.

The architecture of CNN classification model is shown in **Table S1**. During training, validation and testing, the size of each input image is adjusted to 100×100. The loss function of the classifier is defined as cross entropy, and the constructed CNN network uses the Adam optimizer to achieve optimization. The model is initially evaluated and the hyperparameters are optimized by calculating the loss (cross entropy)

and prediction accuracy of the validation set. The hyperparameters of the obtained optimal CNN classification model are as follows: the training set epoch is set to 100, the learning rate is set to 0.0001, and the batch size of the training set and the verification set is 65. The architecture of ANN classification model is shown in **Table S3**. After optimization, the hyperparameter is the learning rate of 0.0002, the batch size of the training set and the verification set is 120, and a total of 1000 epochs are trained. The parameters of ML classification model are summarized in **Table S4**.

7.Quantification modeling

Two deep learning algorithms (CNN, ANN) and six machine learning algorithms (DT, KNN, LR, RF, SVM, XGBoost model) were used to construct the regression model. For details, see **Tables S4 and S9-10**.

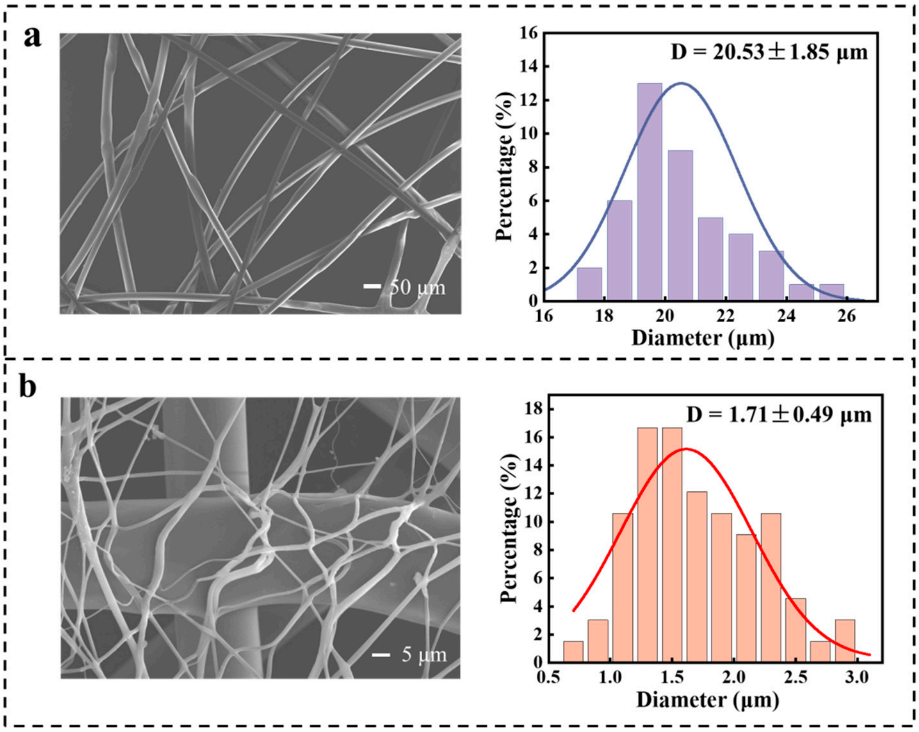

Figure S1. SEM and particle size distribution of 10 g/m^2 non-woven fabric (a) and 50 min TPU electrospinning (b).

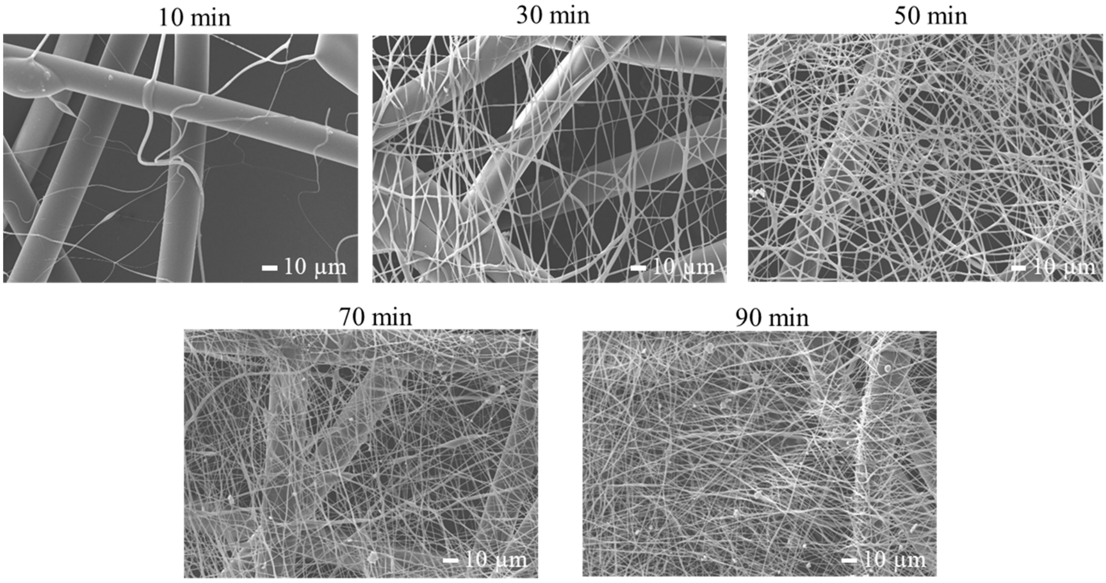

Figure S2. SEM images of different electrospinning time on non-woven fabric.

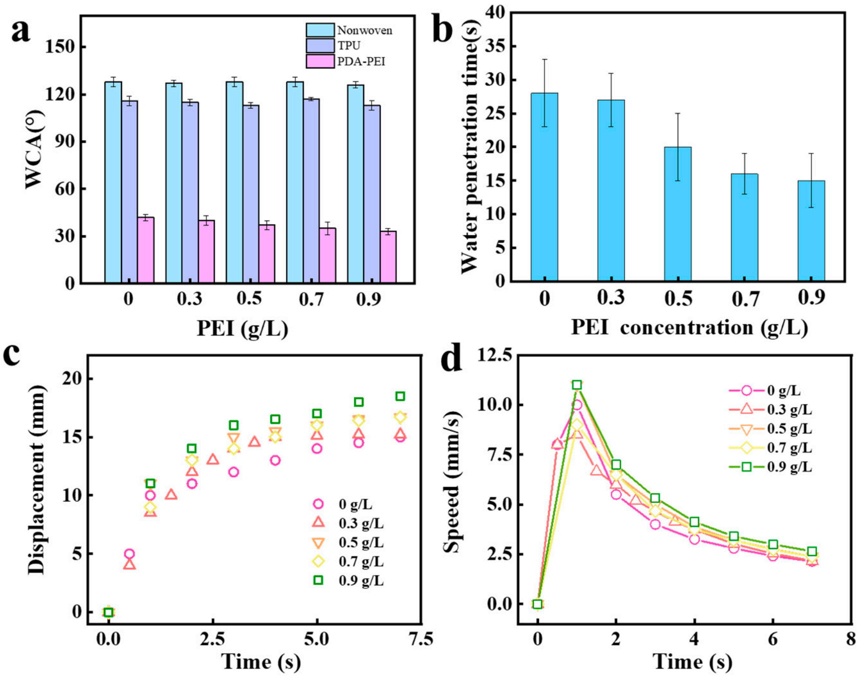

Figure S3. The change of contact Angle of the three layers after deposition of different PEI concentrations (a), the time for 5 μL water droplets to reach the surface of the nonharmful fabric layer until the contact Angle decreases to zero (b), and the time-displacement diagram (c) and velocity diagram (d) for 10 μL water droplets to move from the conical tip to the wide end.

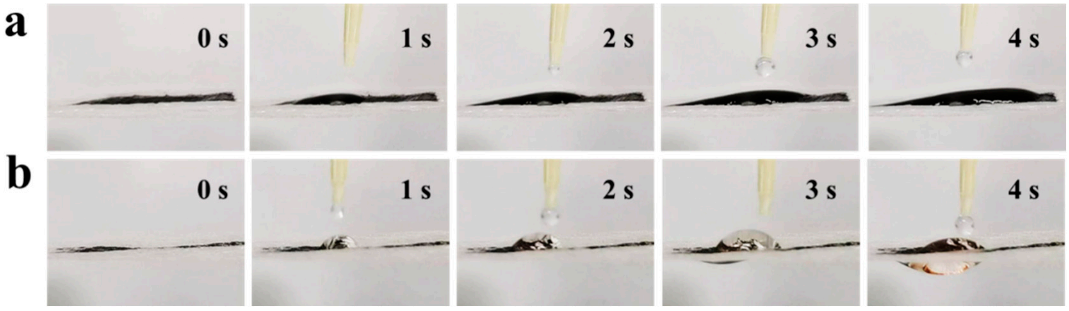

Figure S4. Water droplets are transported from the hydrophilic layer (a) and the hydrophobic layer (b).

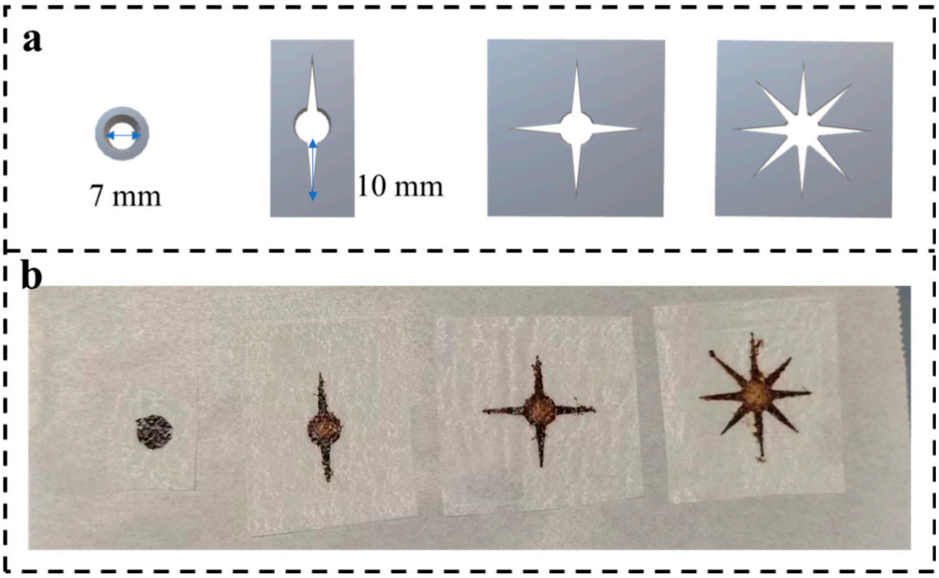

Figure S5. (a) Schematic drawings of patterned molds for circles and different numbers of cones. (b) Schematic drawings of patterned Janus membranes with circles and different numbers of cones.

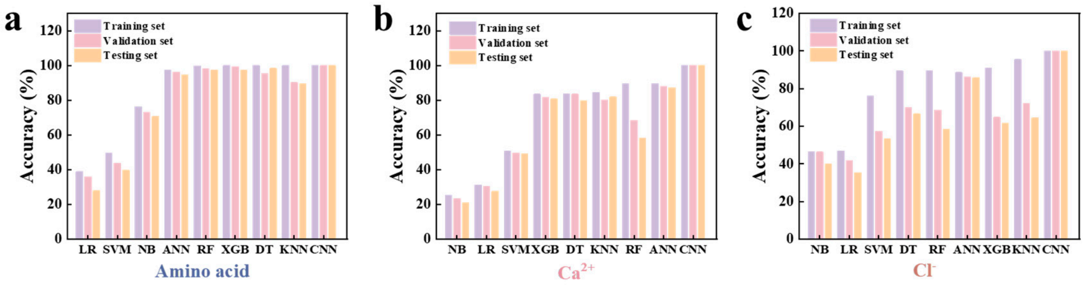

Figure S6. Different classification models (including ANN, XGBoost, DT, KNN, LR, NB, RF, SVM and CNN) were used to compare the prediction accuracy of sweat biomarkers (amino acid mixture, Ca^{2+}, and Cl^{-}) on the training set, validation set and testing set.

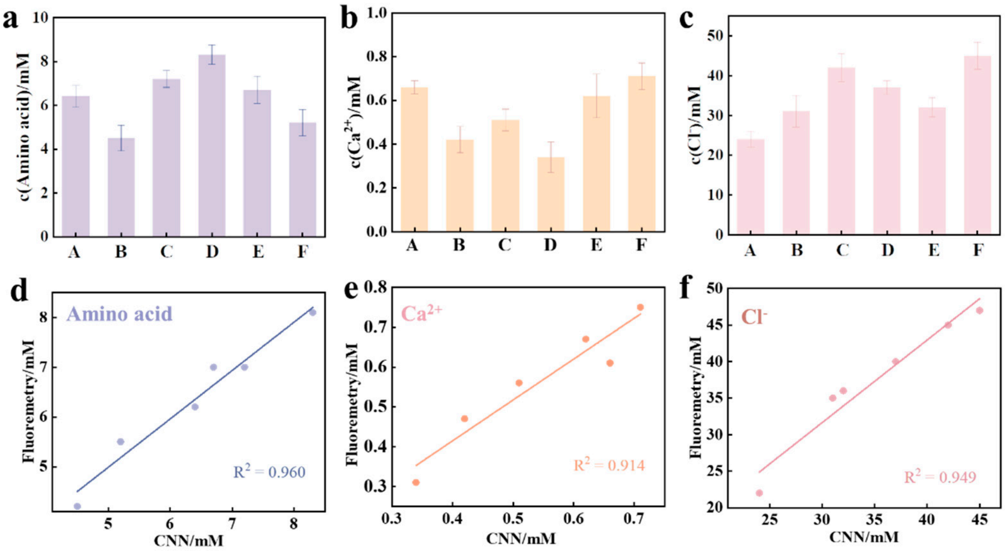

Figure S7. Quantitative determination of amino acid mixture, Ca^{2+} and Cl^- in actual sweat of six subjects during exercise. (a-c) Prediction of amino acid mixture, Ca^{2+}, and Cl^- concentrations by DL-assisted programmable self-calibrating fluorescent patches. (d-f) Evaluation of the accuracy of prediction results of CNN-assisted fluorescent patches compared to fluorescence spectral detection results.

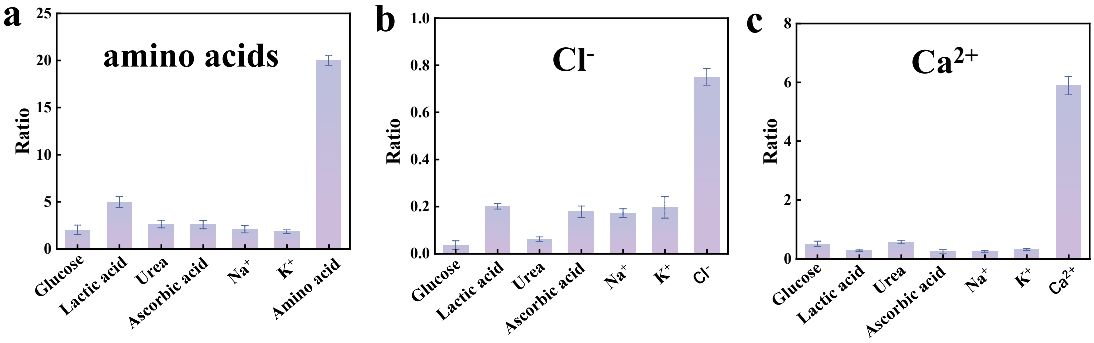

Figure S8. Selectivity of (a) amino acid mixture, (b) Cl^-, and (c) Ca^{2+} in programmable fluorescent hydrogel chips.

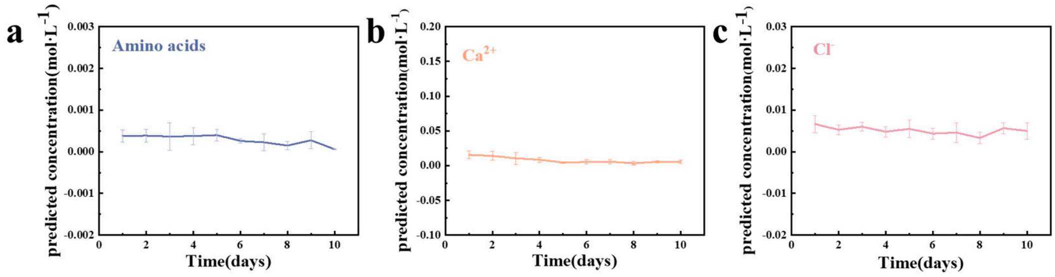

Figure S9. Stability of (a) amino acid mixture, (b) Ca^{2+}, and (c) Cl^- in programmable fluorescent hydrogel chips.

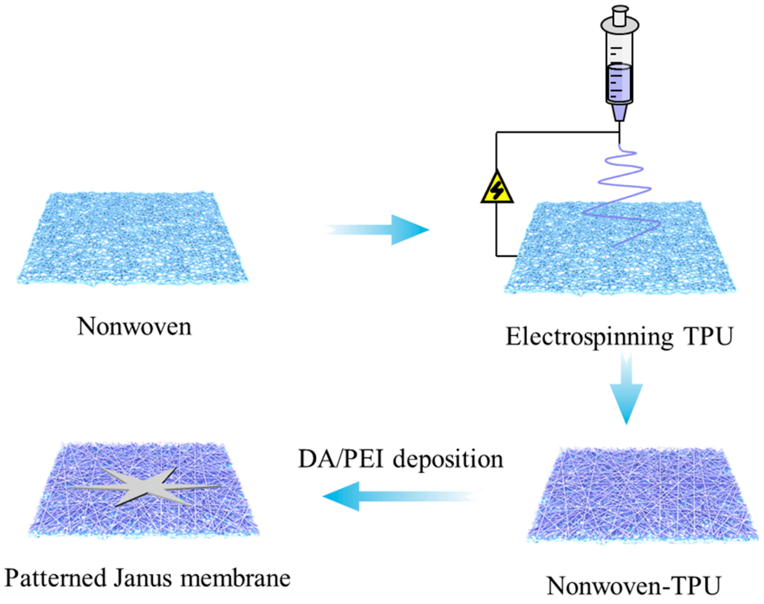

Figure S10. Spontaneous in situ sweat collection patterned Janus membrane preparation.

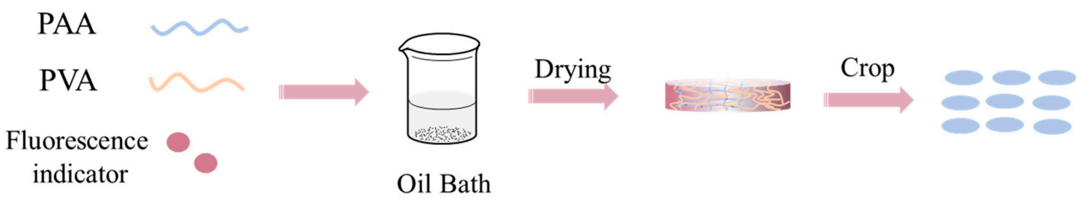

Figure S11. Preparation process of wearable programmable fluorescent hydrogel patch.

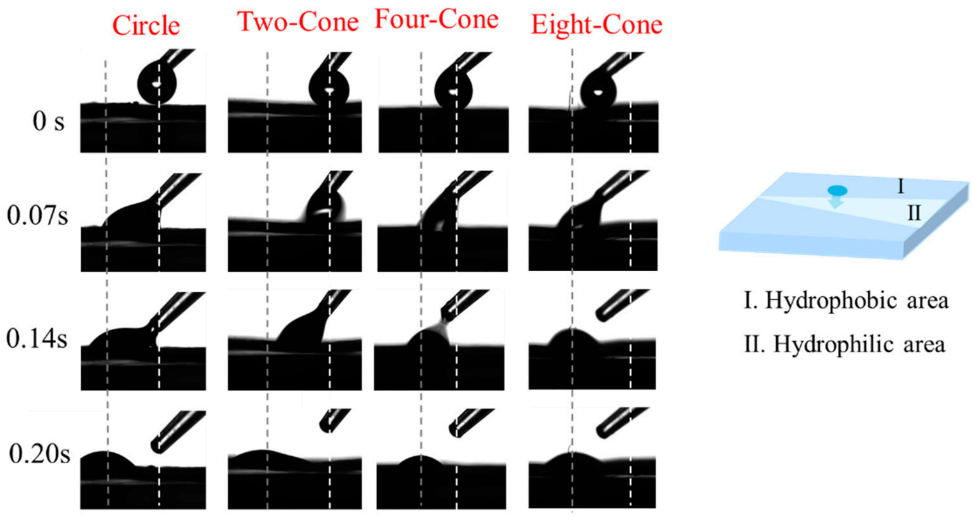

Figure S12. Diagram of 5 μL water droplets moving from the hydrophobic and hydrophilic boundaries of the patterned Janus membrane with circles and different numbers of cones.

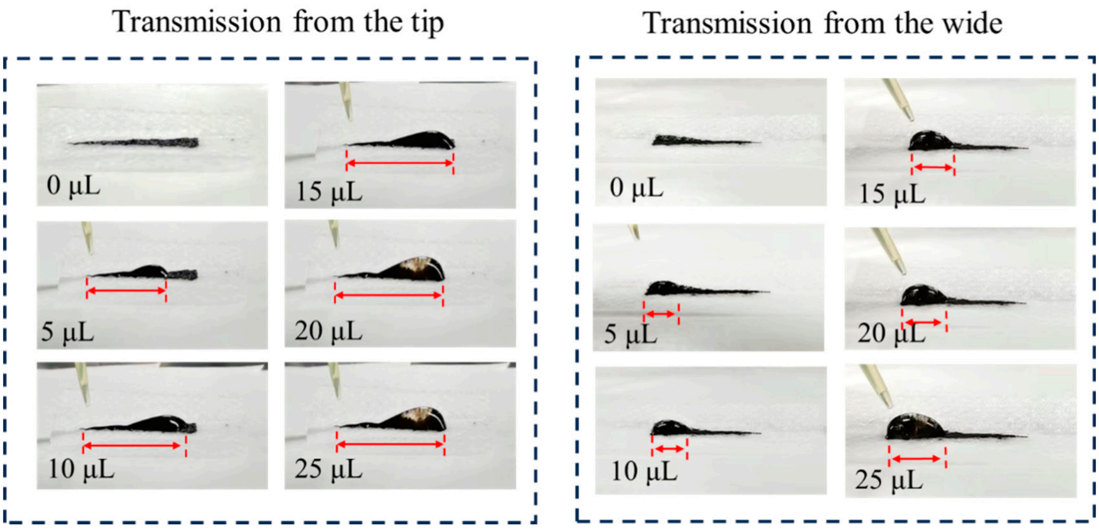

Figure S13. Water droplets are transported from the conical tip and wide end.

Table S1. The architecture of the CNN classification model.

Layer	Layer (type)	Output Shape
**1**	Input layer	(None, 100, 100, 3)
**2**	Convolution layer	(None, 98, 98, 32)
**3**	Max pooling layer	(None, 49, 49, 32)
**4**	Convolution layer	(None, 47, 47, 64)
**5**	Max pooling layer	(None, 23, 23, 64)
**6**	Convolution layer	(None, 21, 21, 128)
**7**	Max pooling layer	(None, 10, 10, 128)
**8**	Convolution layer	(None, 8, 8, 128)
**9**	Max pooling layer	(None, 4, 4, 128)
**10**	Flatten layer	(None, 2048)
**11**	Fully-connected layer	(None, 128)
**12**	Dropout layer	(None, 128)
**13**	Fully-connected layer	(None, 20 or 6)
**14**	Output	(None, 20 or 6)

Table S2. Classification results of DL and ML methods

Amino acid mixture (%)									
	**XGB**	**DT**	**KNN**	**LR**	**RF**	**SVM**	**NB**	**ANN**	**CNN**
Training set	100	100	100	38.8	99.8	49.5	76.3	97.4	100
Validation set	99.4	95.5	90.1	35.9	98.2	43.7	73.1	96.1	100
Testing set	97.3	98.4	89.4	27.9	97.4	39.7	70.5	94.7	100
**Cl^- (%)**									
	**XGB**	**DT**	**KNN**	**LR**	**RF**	**SVM**	**NB**	**ANN**	**CNN**
Training set	83.5	83.8	84.5	31.1	89.7	50.9	25.1	89.5	100
Validation set	81.5	83.5	80.3	30.5	68.5	49.5	23.5	87.9	100
Testing set	80.7	79.7	81.9	27.5	58.3	49.0	21.0	87.3	100
**Ca^{2+} (%)**									
	**XGB**	**DT**	**KNN**	**LR**	**RF**	**SVM**	**NB**	**ANN**	**CNN**
Training set	90.9	89.4	95.5	47.0	89.6	76.2	46.6	88.6	100
Validation set	64.8	70.0	72.2	41.6	68.5	57.4	46.3	86.2	100
Testing set	61.6	66.6	64.4	35.2	58.3	53.3	40.0	85.8	100

Table S3. The architecture of the ANN classification model.

Layer	Layer (type)	Output Shape
1	Input layer	(None, 3)
2	Fully-connected layer	(None, 64)
3	Fully-connected layer	(None, 256)
4	Fully-connected layer	(None, 128)
5	Dropout layer	(None, 64)
6	Fully-connected layer	(None, 20 or 6)
7	Output	(None, 20 or 6)

Table S4. Parameters of ML classification models.

Algorithm	Parameters
XGB	(num_round = 360, eta = 0.07, max_dept = 4, subsample = 0.65)
Decision Trees	(criterion = 'gini', splitter = 'best', max_depth = 25, min_samples_leaf = 3, min_samples_split = 1)
KNN	(n_neighbors = 3, weights = 'uniform', algorithm = 'auto', leaf_size = 30, p = 2, metric = 'minkowski')
Logistic Regression	(solver = 'sag', penalty = '10', C = 14, max_iter = 540)
Naive Bayes	(num_round = 300, criterion = 'gini', gamma = 2.45, subsample = 0.95)
Random Forest	(criterion = 'gini', n_estimators = 46, max_depth =20, min_samples_leaf = 1, min_samples_split = 1)
SVM	(kernel = 'rbf', gamma = 1.74, C = 2.55)

Table S5. The architecture of the CNN quantification model.

**Layer**	**Layer (type)**	**Output Shape**
1	Input layer	(None, 100, 100, 3)
2	Convolution layer	(None, 98, 98, 32)
3	Max pooling layer	(None, 49, 49, 32)
4	Convolution layer	(None, 47, 47, 64)
5	Max pooling layer	(None, 23, 23, 64)
6	Convolution layer	(None, 21, 21, 128)
7	Max pooling layer	(None, 10, 10, 128)
8	Convolution layer	(None, 8, 8, 128)
9	Max pooling layer	(None, 4, 4, 128)
10	Flatten layer	(None, 2048)
11	Fully-connected layer	(None, 128)
12	Dropout layer	(None, 128)
13	Fully-connected layer	(None, 1)
14	Output	(None, 1)

Table S6. Quantitation results of DL and ML methods (Amino acid mixture)

**Amino acid mixture**								
	**Training set**							
	**XGB**	**DT**	**KNN**	**LR**	**RF**	**SVM**	**ANN**	**CNN**
R^2	0.999	0.602	0.928	0.919	0.976	0.821	0.978	0.999
MSE	0.220	2.890	0.554	0.656	0.572	1.114	0.016	0.0
RMSE	0.148	1.764	0.744	0.810	0.756	1.070	0.129	0.017
MAE	0.975	0.976	0.436	0.488	0.658	0.645	0.075	0.014
	**Validation set**							
	XGB	DT	KNN	LR	RF	SVM	ANN	CNN
R^2	0.979	0.651	0.942	0.900	0.986	0.854	0.987	0.999
MSE	0.496	2.564	0.481	0.769	0.325	1.082	0.009	0.001
RMSE	0.709	1.627	0.694	0.877	0.570	1.046	0.098	0.023
MAE	0.681	0.922	0.407	0.550	0.254	0.945	0.064	0.018
	**Testing set**							
	XGB	DT	KNN	LR	RF	SVM	ANN	CNN
R^2	0.988	0.557	0.925	0.876	0.967	0.842	0.979	0.999
MSE	0.469	3.012	0.558	0.935	0.795	0.835	0.016	0.0
RMSE	0.685	1.772	0.747	0.967	0.892	0.914	0.128	0.021
MAE	0.305	1.061	0.422	0.591	0.349	0.845	0.074	0.016

Table S7. Quantitation results of DL and ML methods (Cl^-)

Cl^-								
	**Training set**							
	**XGB**	**DT**	**KNN**	**LR**	**RF**	**SVM**	**ANN**	**CNN**
R^2	0.999	0.819	0.814	0.592	0.592	0.870	0.991	0.999
MSE	0.361	1.407	1.537	2.822	2.208	0.893	0.144	0.0
RMSE	0.638	1.118	1.240	1.686	1.486	0.954	0.380	0.018
MAE	0.424	0.555	0.529	0.841	0.743	0.451	0.184	0.014
	**Validation set**							
	XGB	DT	KNN	LR	RF	SVM	ANN	CNN
R^2	0.958	0.803	0.817	0.593	0.518	0.832	0.980	0.999
MSE	0.665	1.374	1.416	2.496	2.140	1.110	0.159	0.0
RMSE	0.815	1.172	1.119	1.584	1.463	1.054	0.399	0.02
MAE	0.428	0.710	0.508	0.791	0.731	0.546	0.199	0.015
	**Testing set**							
	XGB	DT	KNN	LR	RF	SVM	ANN	CNN
R^2	0.943	0.742	0.689	0.585	0.524	0.842	0.979	0.999
MSE	0.883	1.753	1.939	2.022	2.019	1.214	0.136	0.0
RMSE	0.939	1.324	1.392	1.422	1.421	1.102	0.368	0.02
MAE	0.468	0.662	0.690	0.787	0.710	0.541	0.181	0.017

Table S8. Quantitation results of DL and ML methods (Ca^{2+})

Ca^{2+}								
	**Training set**							
	**XGB**	**DT**	**KNN**	**LR**	**RF**	**SVM**	**ANN**	**CNN**
R^2	0.994	0.482	0.427	0.768	0.946	0.943	0.997	0.999
MSE	0.293	4.397	4.744	1.572	0.328	0.346	0.212	0.001
RMSE	0.542	2.097	2.178	1.254	0.572	0.588	0.460	0.025
MAE	0.275	1.319	1.498	0.627	0.379	0.382	0.313	0.02
	**Validation set**							
	XGB	DT	KNN	LR	RF	SVM	ANN	CNN
R^2	0.953	0.434	0.417	0.714	0.932	0.927	0.987	0.999
MSE	0.269	4.780	4.959	1.651	0.392	0.418	0.320	0.0
RMSE	0.519	2.186	2.222	1.285	0.626	0.646	0.565	0.022
MAE	0.320	1.442	1.512	0.642	0.406	0.408	0.403	0.018
	**Testing set**							
	XGB	DT	KNN	LR	RF	SVM	ANN	CNN
R^2	0.959	0.336	0.370	0.719	0.933	0.936	0.981	0.999
MSE	0.248	4.460	5.347	1.898	0.411	0.392	0.240	0.001
RMSE	0.498	2.111	2.312	1.378	0.641	0.626	0.490	0.024
MAE	0.324	1.387	1.596	0.689	0.453	0.424	0.331	0.019

Table S9. The architecture of the ANN quantification model.

Layer	Layer (type)	Output Shape
1	Input layer	(None, 3)
2	Fully-connected layer	(None, 64)
3	Fully-connected layer	(None, 256)
4	Fully-connected layer	(None, 128)
5	Dropout layer	(None, 64)
6	Fully-connected layer	(None, 1)
7	Output	(None, 1)

Table S10. Parameters of ML quantification models.

**Algorithm**	**Parameters**
XGBoost	(num_round = 100, eta = 0.15, max_dept = 7, subsample = 0.70)
Decision Trees	(criterion = 'absolute_error', splitter = 'best', max_depth = 16, min_samples_leaf = 1, min_samples_split = 2)
KNN	(n_neighbors = 1, weights = 'uniform', algorithm = 'auto', leaf_size = 30, p = 2, metric = 'minkowski')
Random Forest	(criterion = 'absolute_error', n_estimators = 1, max_depth = 17, min_samples_leaf = 1, min_samples_split = 4)
SVM	(kernel = 'rbf', gamma = 1.00, C = 761)
